# Screening of Attention-Deficit Hyperactivity Disorder and its associated factors among medical university students in Lebanon: A multicenter cross-sectional study

**DOI:** 10.1371/journal.pgph.0006528

**Published:** 2026-09-08

**Authors:** Omar Al Jassem, Mariam Darwish, Rami Rifi, Chirine Chawiche, Hassan Khalil, Sara Ayoub, Mabelle Al Bassar, Ayman El Moubayed, Layal Malli, Madeeha Kamal, Linda Abou-Abbas

**Affiliations:** 1 Faculty of Medical Sciences, Lebanese University, Beirut, Lebanon; 2 Faculty of Medical Sciences, Neuroscience Research Center, Lebanese University, Beirut, Lebanon; 3 AZM Center for Research in Biotechnology and its Applications, Doctoral School for Sciences and Technology, Lebanese University, Tripoli, Lebanon; 4 Department of Medicine, University of Missouri, Columbia, Missouri, United States of America; 5 Faculty of Medicine, American University of Beirut Medical Center, Beirut, Lebanon; 6 Neurology Department, American University of Beirut Medical Center, Beirut, Lebanon; 7 Faculty of Medicine, Saint Joseph University of Beirut, Beirut, Lebanon; 8 Gilbert and Rose-Marie Chagoury School of Medicine, Lebanese American University, Byblos, Lebanon; 9 Department of Pediatrics, Division of Adolescent, Sidra Medicine, Doha, Qatar; 10 Weill Cornell Medicine-Qatar (WCM-Q), Weill Cornell University, Doha, Qatar; 11 College of Medicine, Qatar University, Doha, Qatar; 12 INSPECT-LB, Institut National de Santé Publique, Epidémiologie Clinique et Toxicologie, Faculty of Public Health, Lebanese University, Fanar, Lebanon; PLOS: Public Library of Science, UNITED STATES OF AMERICA

## Abstract

Attention-Deficit Hyperactivity Disorder (ADHD) is a common neurodevelopmental disorder that may adversely affect academic performance and well-being. Data on ADHD among Lebanese medical students are limited. This study assessed the prevalence of probable ADHD symptoms and associated factors among medical students in Lebanon. A multicenter cross-sectional study was conducted among medical students from six Lebanese universities. Participants completed an online questionnaire including the Adult ADHD Self-Report Scale (ASRS v1.1), Patient Health Questionnaire-9 (PHQ-9), Generalized Anxiety Disorder-7 (GAD-7), and Pittsburgh Sleep Quality Index (PSQI). Factors associated with ADHD screening scores were identified using multivariable linear regression. A total of 306 medical students participated; 60.8% were female, and the mean age was 21.9 ± 2.1 years. Overall, 93 students screened positive for probable ADHD symptoms, corresponding to a prevalence of 30.4% (95% CI: 25.5%–35.8%). Higher ADHD screening scores were independently associated with fourth academic year (β = 0.605, 95% CI: 0.111–1.099; p = 0.017), middle socioeconomic status (β = 0.399, 95% CI: 0.022–0.777; p = 0.038), comorbidities (β = 0.624, 95% CI: 0.082–1.167; p = 0.024), birth complications (β = 0.888, 95% CI: 0.311–1.466; p = 0.003), smoking (β = 1.054, 95% CI: 0.415–1.692; p = 0.001), higher PHQ-9 scores (β = 0.089, 95% CI: 0.042–0.136; p < 0.001), and poorer sleep quality (PSQI score; β = 0.093, 95% CI: 0.029–0.157; p = 0.005). Nearly one-third of Lebanese medical students screened positive for probable ADHD symptoms. These findings suggest that ADHD symptoms may coexist with modifiable psychological and lifestyle factors, highlighting the need for integrated screening and support services within medical schools. Future longitudinal studies using clinical diagnostic assessments are warranted to clarify causal relationships and confirm ADHD diagnoses.

## Introduction

Attention-Deficit Hyperactivity Disorder (ADHD) is a neurodevelopmental disorder characterized by persistent patterns of inattention and/or hyperactivity and impulsivity that begin in childhood, last for at least six months, and interfere with academic, occupational, or social functioning. ADHD may present as predominantly inattentive, predominantly hyperactive/impulsive, or combined presentation [1]. The mean age of diagnosis is approximately six to seven years [2]. Although traditionally considered as a childhood disorder, many individuals remain undiagnosed until adolescence or adulthood, particularly those with predominantly inattentive symptoms or less overt clinical presentation [3].

Notably, in the Middle East and North Africa (MENA) region, ADHD prevalence varies across countries and age groups. A recent systematic review and meta-analysis conducted in 2024 including 849 902 participants reported ADHD prevalence rates ranging from 1.3% in Yemen to 22.2% in Iran, with pooled estimates of 13.5% in adults and 10.1% in children and adolescents [4]. In Lebanon, a study among school-age children reported prevalence rates of 3 per 1000 for the inattentive subtype, 12 per 1000 for the hyperactive-impulsive subtype, and 17 per 1000 for the combined subtype [5]. Another Lebanese study among adolescents reported a 30-day prevalence of 10.2% [6].

ADHD is associated with substantial long-term morbidity when left untreated [7]. Individuals with ADHD are at increased risk of poor academic achievement, impaired occupational performance, and difficulties in social and interpersonal functioning [8]. It is also strongly associated with psychiatric comorbidities such as depression, anxiety disorders, and substance use disorders [9,10], as well as increased risk of accidental injuries and risky behaviors [11]. These consequences often persist into adulthood and can significantly reduce quality of life and functional outcomes, particularly in high-demand academic environments such as medical education [12].

ADHD is a multifactorial disorder influenced by genetic, prenatal, and postnatal factors. Genetics play a major role, with heritability estimated at approximately 74% in twin studies [13]. Prenatal factors, including maternal stress, toxin exposure (e.g., nicotine and alcohol), infections, anemia, and vitamin deficiencies, have been implicated in increased risk [14–17]. Postnatal factors include vitamin D deficiency, acquired brain injury, obesity, mineral imbalances, genetic variants such as Monoamine Oxidase A, and childhood maltreatment [18–20].

ADHD is frequently associated with neurodevelopmental and psychiatric comorbidities, including autism spectrum disorder, learning disorders, tic disorders, mood and anxiety disorders, conduct disorder, oppositional defiant disorder, substance use disorder, sleep problems, epilepsy, and post-traumatic stress disorder [19,21]. This clinical complexity complicates diagnosis and highlights the importance of early identification and management to improve long-term outcomes [22].

Regarding university students in the MENA region, several studies report high rates of probable ADHD symptoms. In the United Arab Emirates, a cross-sectional study reported a prevalence of 34.7% among university students [23], while another study reported 13.6% [23,24]. In Saudi Arabia, 26% of medical students screened positive for ADHD symptoms [25]. In Pakistan, prevalence ranged from 12.17% to 34.8% across different studies [26,27], with associations including inattentive symptoms, screen time, and physical inactivity.

Globally, efforts to improve ADHD recognition and management have included the development of standardized diagnostic criteria, clinical practice guidelines, school- and university-based screening initiatives, and increased integration of mental health services into primary care and educational settings [28]. Despite these advances, ADHD remains underdiagnosed, particularly in adolescents and young adults, due to limited awareness, stigma, and variability in access to mental health services [29]. In the MENA region, most research and interventions have focused on children and adolescents, with fewer structured university-based screening or intervention programs.

In Lebanon, previous studies have assessed ADHD among children and adolescents [5,30] and explored familial risk factors and barriers to care [31,32]. However, research focusing on university students, particularly medical students, remains limited. This is important because medical students are exposed to high academic demands, sleep disruption, and psychological stress, which may exacerbate attentional difficulties and delay recognition of symptoms [33]. To our knowledge, no previous Lebanese study has specifically screened for ADHD symptoms among medical students while simultaneously examining sociodemographic, prenatal, lifestyle, and psychological correlates. Therefore, this study aimed to screen for ADHD symptoms and identify associated factors among medical students in Lebanon.

## Methods

### Study design and setting

This study was a cross-sectional, descriptive, web-based survey conducted in Lebanon between 26 November 2025 and 20 December 2025. The source population comprised students from six Lebanese universities: Lebanese University, American University of Beirut, Lebanese American University, Beirut Arab University, Saint Joseph University of Beirut, and the university of Balamand.

### Participants and eligibility criteria

The study population were medical students aged 18 years or older, residing in Lebanon, enrolled in one of the participating universities during the study period, and willing to provide electronic informed consent. Students from all academic years were included. Participants who declined consent or submitted incomplete questionnaires were excluded from the analysis.

### Sample size

The sample size has been calculated using online sample size calculation program with a 5% margin of error, a 95% confidence level, an estimated response distribution of 20%, and with 222,000 registered university students in Lebanon. it was estimated that 246 participants will be required as minimum in order to achieve the objectives of the study. A total of 306 medical students were ultimately recruited, exceeding the minimum required sample size.

### Sampling technique

A non-probability snowball sampling method was used to recruit participants. This approach was selected because it allowed rapid dissemination of the survey across multiple universities and academic years in the absence of a complete accessible sampling frame of Lebanese medical students. One or two medical students from each of the six participating universities distributed the questionnaire through medical student networks and social media platforms within their respective medical schools across different academic years.

### Data collection instrument

Data collection was conducted electronically through a self-administered online questionnaire created using Google Forms with all items marked as required and composed of three main sections. The first section includes:

1. sociodemographic and academic characteristics: gender, age, marital status, governorate of residency, university, academic year, clinical rotation status, socioeconomic status, living arrangements, maternal history during pregnancy (e.g., smoking status, age at delivery, mode of delivery, NICU admission), and personal history of chronic or mental health disorders.
2. Lifestyle Factors: Sleep duration, exercise habits, smoking and alcohol consumption, daily study hours, and time spent on smartphone or other electronic devices.

The second section includes: Adult ADHD Self-Report Scale (ASRS v1.1) symptom checklist part A, a validated 6-item screening tool assessing ADHD symptoms in adults over the past 6 months rather than lifetime ADHD diagnosis. Each item is rated on a 5-point Likert scale. For the first 3 items, responses of “Sometimes”, “Often” or “Very often” are scored 1, and “Never” or “Rarely” are scored 0. For the last 3 items, responses of “Often” or “Very Often” are scored 1, and all other responses are scored 0. A total score of 4 or more indicated positive screening for probable ADHD symptoms and the need for further clinical evaluation [34].

The third section assessed other psychiatric and mental health comorbidities and contained:

1. Patient Health Questionnaire (PHQ-9), a validated 9-item self-report scale designed to assess depression. Each item is rated from 0 (Not at all) to 3 (Nearly every day), giving a total score range of 0–27. The depression severity is considered to be: none/minimal for a score of 0–4, mild for a score of 5–9, moderate for a score of 10–14, moderately severe for a score of 15–19, and severe for a score of 20–27 [35].
2. Generalized Anxiety Disorder (GAD-7), a validated 7-item self-reported questionnaire for screening and measuring the severity of GAD according to reported response categories with assigned points that range from 0 (not at all sure) to 3 (nearly every day). The total score is the sum of all items, with higher values representing greater severity of symptoms. The GAD-7 scores range from 0 to 21. The severity of GAD is considered to be: absent or low risk for a score of 0–4, mild for a score of 5–9, moderate for a score of 10–14, and severe for a score ≥ 15 [36].
3. Pittsburgh Sleep Quality Index (PSQI), a validated self-report questionnaire used to assess subjective sleep quality and disturbances over a one-month time interval. It consists of 19 items from which a global score and seven component scores can be derived. The component scores include: subjective sleep quality, sleep latency, sleep duration, habitual sleep efficiency, sleep disturbances, use of sleeping medications, and daytime dysfunction. Each component is scored on a scale from 0 to 3, where a score of 0 indicates no difficulty, while a score of 3 indicates severe difficulty. The global PSQI score is obtained by summing the seven component scores, yielding a total score ranging from 0 to 21. A higher score reflects poorer sleep quality. A total score greater than 5 has been validated as a sensitive and specific cutoff for distinguishing good from poor sleepers across diverse populations [37]. A pilot study was conducted on 15 medical students to ensure clarity and understandability of the questionnaire.

### Data quality assurance

A pilot study was conducted among 15 medical students to assess questionnaire clarity and comprehensibility. All items in Google Forms were set as mandatory to minimize missing data. Participants were restricted to one submission through Google account sign-in. The dataset was checked for completeness, and inconsistent responses before analysis. Only fully completed questionnaires were included in the final analysis.

### Statistical analysis

The collected data were analyzed using the Statistical Package of the Social Sciences (SPSS) version 25. Descriptive statistics were conducted where continuous variables were expressed as mean ± standard deviation (SD), while categorical variables were presented as numbers and percentages. Continuous variables were assessed for normality using graphical methods and statistical tests. Bivariate analyses (t-test & ANOVA) were conducted to examine differences in ADHD screening scores across participant characteristics. Multivariable linear regression analysis was performed to identify factors independently associated with ADHD screening scores. The ASRS v1.1 provides a continuous score reflecting the severity of ADHD symptoms; therefore, ADHD symptom severity was treated as a continuous outcome variable in the regression model. This approach was chosen to retain variability in symptom severity and to maximize statistical power. Variables with a p-value < 0.20 in bivariate analysis were entered into the multivariable model. Regression coefficients (β), 95% confidence intervals (CI), and p-values were reported. Statistical significance was set at p < 0.05.

### Ethical considerations

This study was conducted following the approval of the Institutional Review Board (IRB) of the Lebanese Hospital Geitaoui–University Medical Center with approval ID number: 2025-IRB-038. This research was conducted in accordance with the Code of Ethics of the World Medical Association (Declaration of Helsinki III). Prior to participating in this web-based survey, participants were presented with a consent form on the first page. This form provided detailed information about the study’s purpose and aim, scope, data usage, privacy measures taken to ensure data confidentiality and anonymity, and data management procedures. Participants were required to read and agree to the terms outlined in the consent form, indicating their voluntary participation and understanding of the study before proceeding further. Respondents were assured of the confidentiality, privacy, and anonymity of their information. Furthermore, participants were under no obligation and had the freedom to withdraw from the study at any point. Consent was obtained through a “Yes/No” question stating: “Do you agree to participate in this study?” All collected data are confidential and are solely accessible by the team for research purposes. As this was an anonymous screening-based survey rather than a diagnostic or interventional study, no individualized clinical follow-up was performed.

## Results

### Participant characteristics

A total of 306 medical students participated in the study. As shown in Table 1, the mean age of the participants was 21.9 ± 2.1 years. Females constituted 60.8% of the sample, and the most were single (79.7%) and living with their families (62.7%). Participants were recruited from six Lebanese universities, with the largest proportion enrolled at the Lebanese University (34.6%), followed by Saint Joseph University of Beirut (17.0%), the American University of Beirut (13.7%), the Lebanese American University (13.1%), Beirut Arab University (12.1%), and the University of Balamand (9.5%).

**Table 1:** Sociodemographic and academic characteristic of the medical students (N = 306).

Variable	n (%)	Mean for ADHD (SD)	p-value
**Age (years) Mean (SD)**	21.9 (2.1)	–	–
**Gender n (%)**			
Female	186 (60.8)	2.72 (1.7)	**0.005**
Male	120 (39.2)	2.14 (1.74)	
**Marital Status n (%)**			
Single	244 (79.7)	2.41 (1.69)	0.162
Engaged / in a relationship	59 (19.3)	2.85 (1.90)	
Married	3 (1)	1.67 (1.53)	
**University n (%)**			
Lebanese University	106 (34.6)	2.34 (1.54)	**0.003**
Saint Joseph University of Beirut (USJ)	52 (17.0)	2.08 (1.87)	
American University of Beirut (AUB)	42 (13.7)	2.1 (1.78)	
Lebanese American University (LAU)	40 (13.1)	2.95 (1.79)	
Beirut Arab University (BAU)	37 (12.1)	3.32 (1.73)	
University of Balamand (UOB)	29 (9.5)	2.66 (1.65)	
**Medical Academic Year (2025–2026) n (%)**			
First year	11 (3.6)	2.36 (2.25)	0.149
Second year	40 (13.1)	2.38 (1.35)	
Third year	37 (12.1)	2.46 (1.83)	
Fourth year	44 (14.4)	3.23 (1.80)	
Fifth year	66 (21.6)	2.35 (1.74)	
Sixth year	59 (19.3)	2.31 (1.84)	
Seventh year	49 (16.0)	2.39 (1.55)	
**Started clinical rotations n (%)**			
No	165 (53.9)	2.57 (1.70)	0.387
Yes	141 (46.1)	2.40 (1.78)	
**Family socioeconomic status n (%)**			
Low (<500 USD/month)	7 (2.3)	2.86 (1.95)	0.169
Middle (500–1000 USD)	91 (29.7)	2.80 (1.69)	
Middle-high (1000–3000 USD)	131 (42.8)	2.39 (1.69)	
High (>3000 USD)	77 (25.2)	2.26 (1.84)	
**Living arrangement n (%)**			
With family	192 (62.7)	2.44 (1.74)	0.815
Dormitory alone	57 (18.6)	2.54 (1.68)	
With roommates	57 (18.6)	2.60 (1.82)	

*Living arrangement was categorized as whether participants lived with their family or resided in a dormitory, either alone or with roommates.

Regarding academic level, students from all seven years of medical training were represented. Fifth-year students constituted the largest group (21.6%), followed by sixth-year (19.3%), seventh-year (16.0%), fourth-year (14.4%), second-year (13.1%), third-year (12.1%), and first-year students (3.6%). Slightly more than half of the participants had not yet started clinical rotations (53.9%).

Regarding the socioeconomic status, 42.8% of the students reported a middle high family income, followed by middle (29.7%) and high socioeconomic status (25.2%), whereas only 2.3% reported low socioeconomic status. Concerning living arrangements, 62.7% lived with their families, while 18.6% lived alone in a dormitory and 18.6% lived with roommates.

Table 2 shows that only 11.4% and 9.8% of the participants reported chronic medical condition and mental health condition, respectively. Moreover, about 13 participants (4.2%) reported currently taking medication for mental health illness. A total of 50.7% of students reported high or extreme levels of academic stress. Regarding prenatal risk factors, maternal smoking during pregnancy was reported by 2% of the participants, while maternal alcohol consumption during pregnancy was not reported by any participants. A maternal medical condition during pregnancy was reported by 6.2% of the participants. Most of the participants were born via vaginal delivery (65%), and only 9.8% have experienced birth complications and 6.5% were admitted to the neonatal intensive care unit. Concerning the lifestyle habits, the majority (84%) reported sleeping between five and eight hours. Moreover, physical activity was reported by 62.4% of the participants, most commonly one to two times per week (39.5%). The majority of the students were non-smoker (89.2%) and only 29.8% consume alcohol. In addition, nearly half of the students reported consuming one to two cups of caffeine per day (49.0%) and smartphone use exceeding five hours per day was reported by 39.9% of participants.

**Table 2:** Clinical, prenatal and lifestyle factors among medical students (N = 306).

Variable	n (%)	Mean for ADHD (SD)	p-value
**Chronic medical condition**			
No	271 (88.6)	2.39 (1.70)	**0.004**
Yes	35 (11.4)	3.29 (1.87)	
**Previously diagnosed mental health condition**			
No	276 (90.2)	2.38 (1.68)	**<0.001**
Yes	30 (9.8)	3.53 (1.94)	
**Currently taking psychiatric medication**			
No	293 (95.8)	2.42 (1.7)	**<0.001**
Yes	13 (4.2)	4 (1.77)	
**Perceived academic stress [1–5]**			
1 (not stressed)	11 (3.6)	1.18 (0.98)	**<0.001**
2	35 (11.4)	1.80 (1.51)	
3	105 (34.3)	2.34 (1.72)	
4	114 (37.3)	2.76 (1.65)	
5 (extremely stressed)	41 (13.4)	3.05 (1.99)	
**Maternal smoking during pregnancy**			
No	292 (95.4)	2.48 (1.74)	0.818
Yes	6 (2.0)	2.50 (1.87)	
I don’t know	8 (2.6)	2.88 (1.89)	
**Maternal medical condition during pregnancy**			
No	259 (84.6)		**0.037**
Yes	19 (6.2)		
I don’t know	28 (9.2)		
**Mode of delivery**			
Vaginal birth	199 (65.0)	2.58 (1.76)	0.229
C-section birth	107 (35.0)	2.33 (1.69)	
**Birth complications**			
No	276 (90.2)	2.42 (1.75)	**0.043**
Yes	30 (9.8)	3.10 (1.47)	
**NICU admission after birth**			
No	286 (93.5)	2.45 (1.75)	0.175
Yes	20 (6.5)	3.00 (1.56)	
**Weekday sleep duration**			
5–8 hours	257 (84.0)	2.92 (1.83)	0.35
<5 hours	27 (8.8)	2.46 (1.72)	
> 8 hours	22 (7.2)	2.27 (1.72)	
**Physical activity**			
None	115 (37.6)	2.72 (1.71)	0.074
1–2 times/week	121 (39.5)	2.48 (1.78)	
3–4 times/week	55 (18.0)	1.98 (1.62)	
Daily	15 (4.9)	2.67 (1.76)	
**Smoking status**			
Non-smoker	273 (89.2)	2.41	**0.016**
Smoker	25 (8.2)	3.44	
Ex-smoker	8 (2.6)	2.25	
**Alcohol consumption**			
Do not drink	215 (70.2)	2.39 (1.73)	0.228
Occasionally	88 (28.8)	2.72 (1.72)	
1–2 cups/day	3 (1.0)	3.33 (3.06)	
**Screen use duration**			
<3 hours	68 (22.2)	2.47 (1.75)	0.644
3-5 hours	116 (37.9)	2.39 (1.73)	
>5 hours	122 (39.9)	2.60 (1.74)	
**Caffeine consumption**			
None	41 (13.4)	2.10 (1.53)	0.168
Rarely	82 (26.8)	2.51 (1.67)	
1–2 cups/day	150 (49.0)	2.49 (1.81)	
3–4 cups/day	30 (9.8)	2,80 (1.73)	
> 5 cups/day	3 (1.0)	4.33 (1.53)	

* Perceived academic stress was self-rated on a 5-point scale ranging from 1 = not stressed to 5 = extremely stressed.

### Prevalence of positvie-ADHD screening among medical students in Lebanon

**Fig 1:**
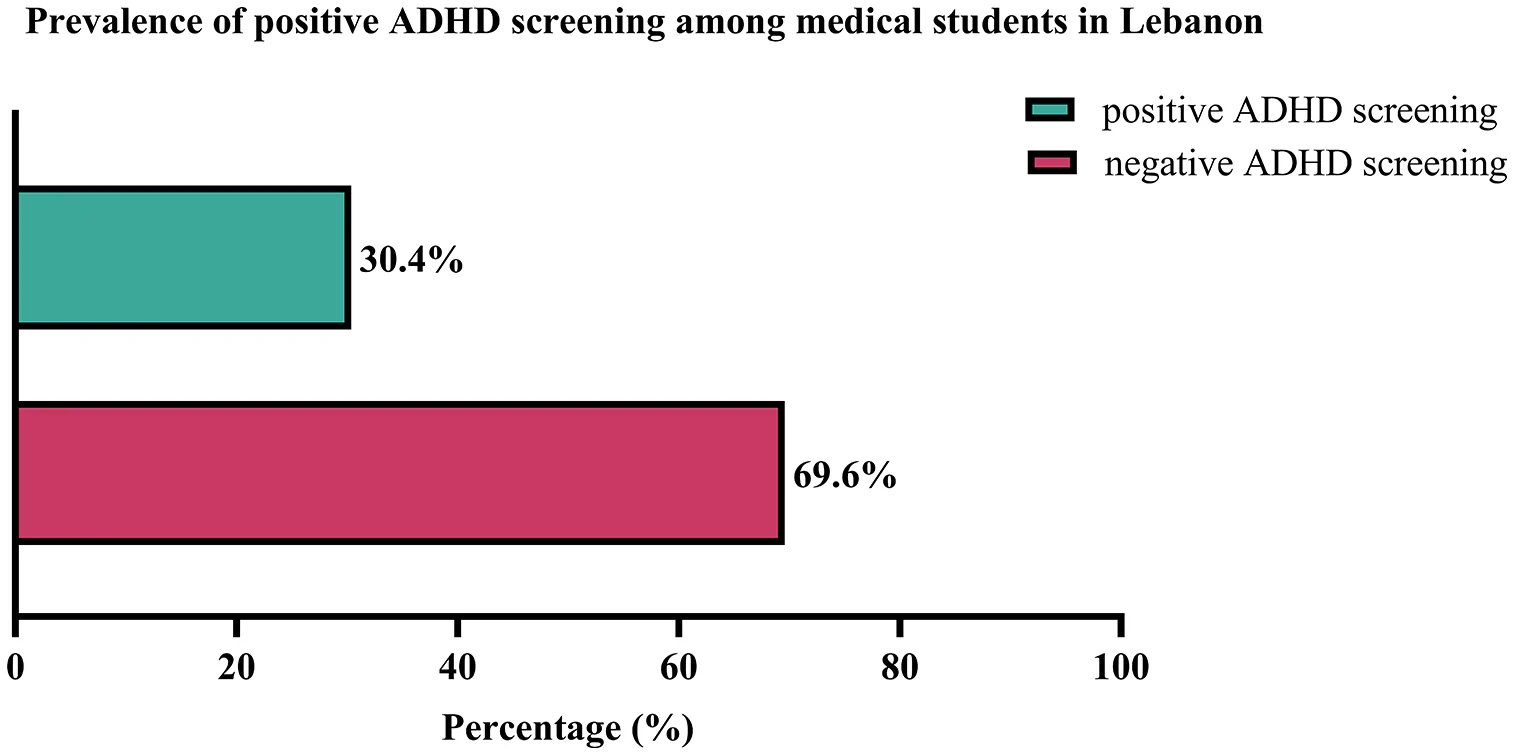
Distribution of ADHD screening status among medical university students.

Overall, 93 out of 306 participants (30.4%; 95% CI: 25.5%–35.8%) screened positive for ADHD symptoms using the ASRS v1.1 as shown in the Fig 1.

#### Association between ADHD screening scores and sociodemographic/academic variables and the clinical, prenatal and lifestyle factors among medical students.

Table 1 presents the bivariate analysis between ADHD screening scores and participants’ sociodemographic and academic characteristics. Female students had significantly higher mean ADHD screening scores compared to males (2.72 ± 1.70 vs. 2.14 ± 1.74, p = 0.005). A significant association was also observed between university affiliation and ADHD screening scores (p = 0.003), with the highest mean scores reported among students from Beirut Arab University (3.32 ± 1.73) and the Lebanese American University (2.95 ± 1.80). No statistically significant associations were found between ADHD screening scores and marital status (p = 0.162), medical academic year (p = 0.149), initiation of clinical rotations (p = 0.387), family socioeconomic status (p = 0.169), or living arrangement (p = 0.815).

As shown in Table 2, students with chronic medical conditions scored higher than those without (3.29 ± 1.87 vs. 2.39 ± 1.70, p = 0.004), and ADHD scores increased progressively with higher levels of perceived academic stress (p < 0.001). Birth complications were also associated with high ADHD scores (3.10 ± 1.47 vs. 2.42 ± 1.75, p = 0.043). Smoking status was significant, with current smokers showing higher scores compared to non-smokers (3.44 ± 1.78 vs. 2.41 ± 1.71, p = 0.016). In addition, students with a previously diagnosed mental health condition (3.53 ± 1.94 vs. 2.38 ± 1.68, p < 0.001) or currently taking psychiatric medication had higher scores than those without (4.0 ± 1.77 vs. 2.42 ± 1.70, p < 0.001).

### Association of ADHD screening status with depression, anxiety, and sleep quality

**Fig 2:**
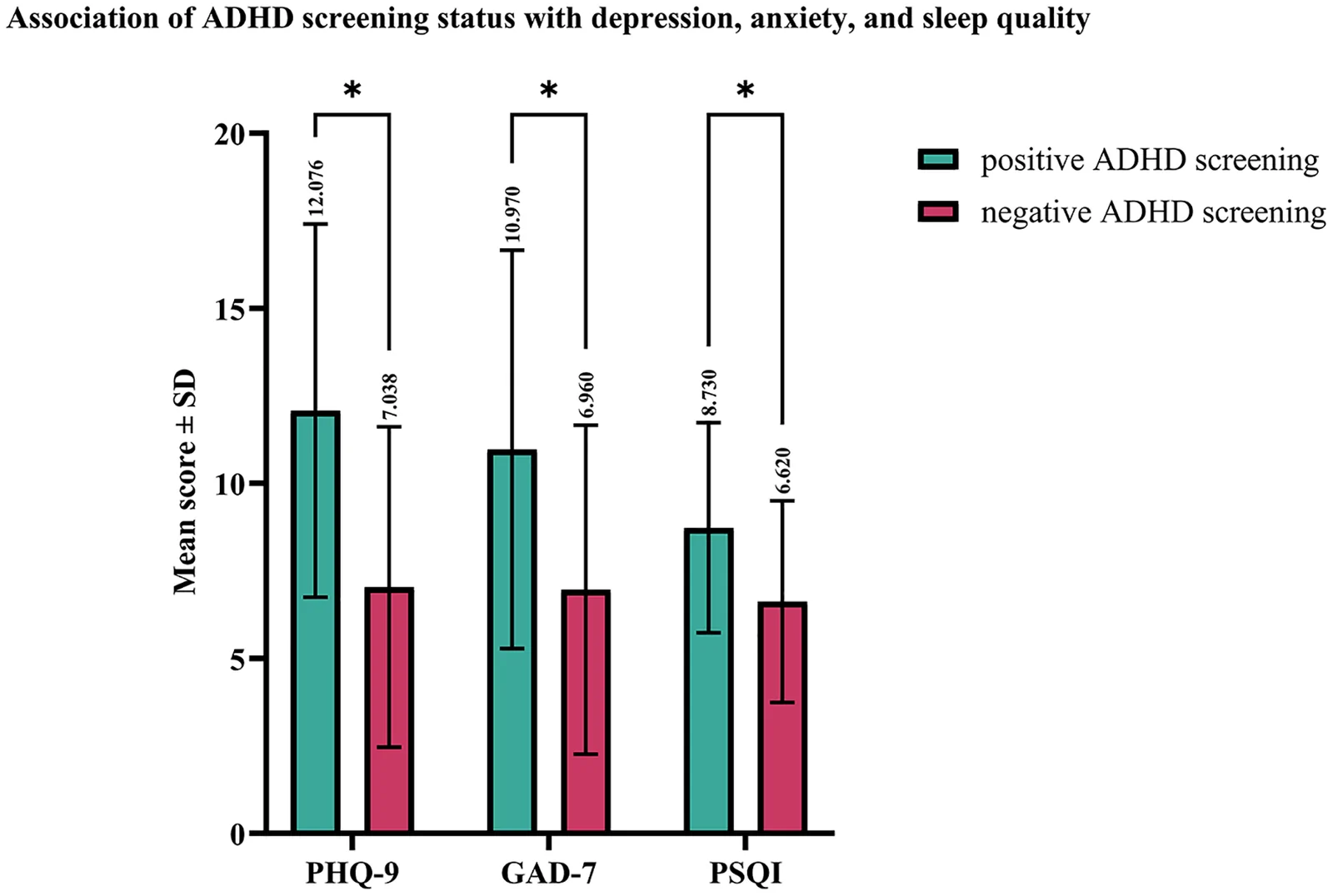
Association of ADHD screening status with depression, anxiety and quality of sleep.

Fig 2 presents the association between ADHD screening status and symptoms of depression, anxiety, and sleep quality. Participants with positive screening for ADHD (n = 93) had significantly higher mean depression scores on the PHQ-9 compared with those without ADHD (12.076 ± 5.33 vs. 7.038 ± 4.58; p < 0.0001). Similarly, anxiety symptoms measured by the GAD-7 were significantly higher in the ADHD positive group than in the non-ADHD group (10.97 ± 5.69 vs. 6.96 ± 4.70; p < 0.001). Moreover, sleep quality, assessed using the PSQI, was also significantly poorer among participants with positive ADHD screening, with higher global PSQI scores compared to those with negative ADHD screening (8.73 ± 3.00 vs. 6.62 ± 2.88; p < 0.001).

**Fig 3:**
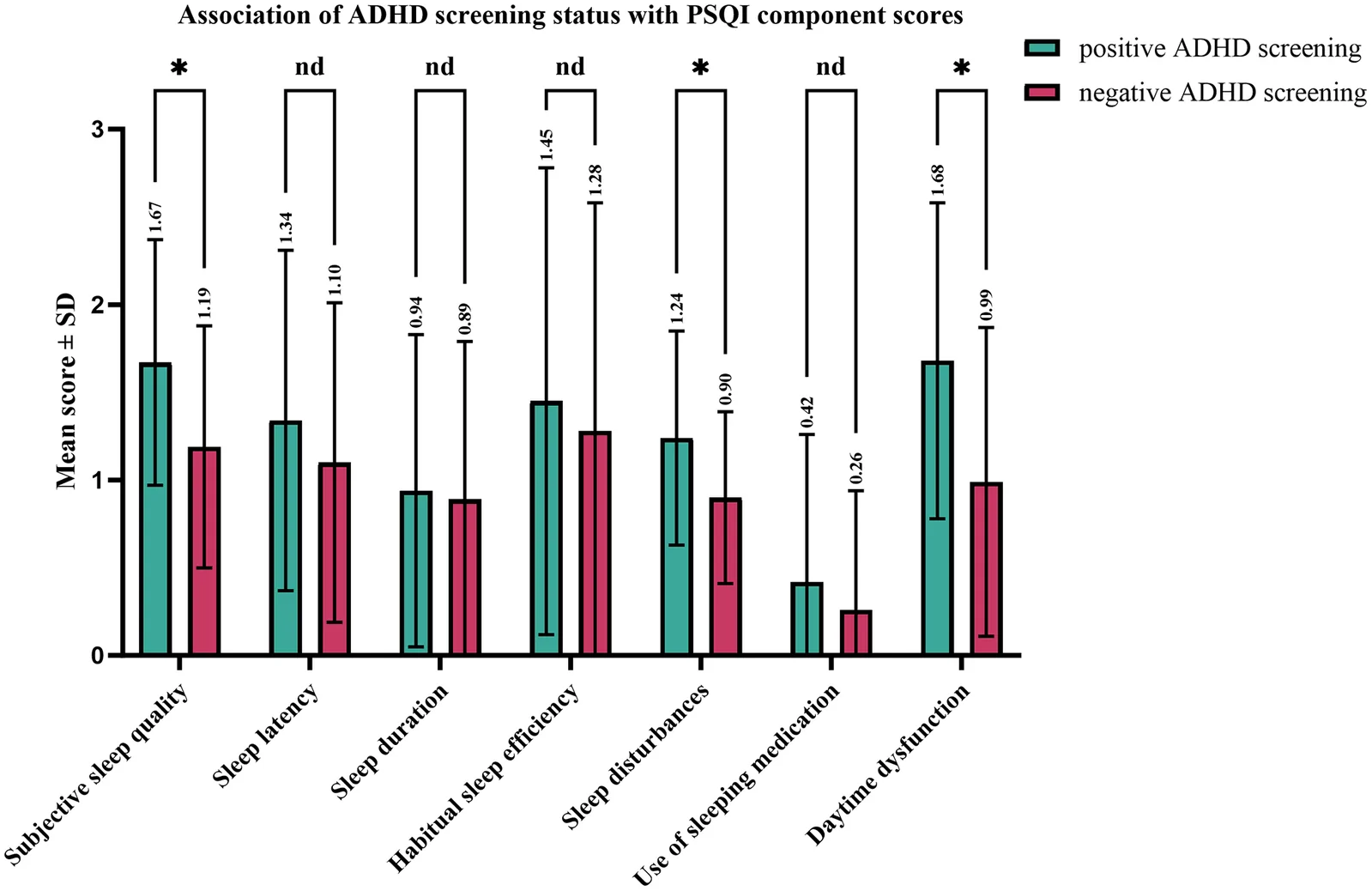
Mean PSQI component scores among participants with positive versus negative ADHD screening.

Fig 3 summarizes the association between ADHD screening status and sleep quality as measured by the PSQI. Significant differences were observed across several PSQI components. Students with ADHD reported worse subjective sleep quality (Component 1: 1.67 ± 0.70 vs. 1.19 ± 0.69; p < 0.001), longer sleep latency (Component 2: 1.34 ± 0.97 vs. 1.10 ± 0.91; *p* = 0.037), greater sleep disturbances (Component 5: 1.24 ± 0.61 vs. 0.90 ± 0.49; p < 0.001), and increased daytime dysfunction (Component 7: 1.68 ± 0.90 vs. 0.99 ± 0.88; p < 0.001). No statistically significant differences were found between groups for sleep duration (Component 3; p = 0.697), habitual sleep efficiency (Component 4; p = 0.298), or use of sleep medication (Component 6; p = 0.076).

### Multivariable analysis of factors associated with ADHD scores

Table 3 presents the multivariable linear regression analysis examining factors independently associated with higher ADHD screening scores. Students in the fourth academic year had significantly higher ADHD scores compared to first year students (Unstandardized β = 0.605, p = 0.017). Participants reporting a middle socioeconomic status also showed higher ADHD scores compared to those with a high socioeconomic status (Unstandardized β = 0.399, p = 0.038).

**Table 3:** Multivariable linear regression analysis of factors associated with ADHD screening scores.

Variables	Unstandardized β	Std. Error	Β	p-value	95% CI (Lower)	95% CI (Upper)
Gender (Male vs Female)	-0.350	0.180	-0.098	0.053	-0.705	0.004
Academic year: Fourth year vs First year	0.605	0.251	0.122	**0.017**	0.111	1.099
Middle socioeconomic status vs High socioeconomic status	0.399	0.192	0.105	**0.038**	0.022	0.777
Comorbidity (Yes vs No)	0.624	0.276	0.112	**0.024**	0.082	1.167
Birth complication (Yes vs No)	0.888	0.293	0.149	**0.003**	0.311	1.466
Smoker vs Ex-smoker	1.054	0.324	0.161	**0.001**	0.415	1.692
PHQ score*	0.089	0.024	0.270	**<0.001**	0.042	0.136
GAD score**	0.020	0.023	0.060	0.399	-0.026	0.065
PSQI score***	0.093	0.033	0.162	**0.005**	0.029	0.157

*Patient Health Questionnaire.

**Generalized Anxiety Disorder score.

***Pittsburgh Sleep Quality Index score.

The presence of comorbidities (Unstandardized β = 0.624, p = 0.024) and a history of birth complications (Unstandardized β = 0.888, p = 0.003) were significantly associated with increased ADHD screening scores. In addition, current smokers had significantly higher ADHD scores compared to ex-smokers (Unstandardized β = 1.054, p = 0.001).

Higher PHQ-9 scores were significantly associated with increased ADHD screening scores (Unstandardized β = 0.089, p < 0.001), indicating a positive association between depressive symptoms and ADHD symptoms. Poor sleep quality, reflected by higher PSQI scores, was also significantly associated with higher ADHD screening scores (Unstandardized β = 0.093, p = 0.005).

## Discussion

This study screened for probable ADHD symptoms and examined associated sociodemographic, clinical, prenatal, lifestyle, and psychological factors among medical students in Lebanon. The main findings indicate that nearly one-third of participants screened positive for probable ADHD symptoms. Higher ADHD symptom scores were significantly associated with psychological distress (depression and anxiety), sleep disturbances, smoking behavior, chronic medical conditions, and prenatal/birth complications. These findings suggest that ADHD symptomatology among medical students is multifactorial and closely intertwined with mental health status, behavioral patterns, and early-life and clinical risk factors.

The prevalence observed in this study is consistent with reports from Saudi Arabia (26%) and Pakistan (34.8%), but higher than estimates from China (3.5%) and the United Arab Emirates (13.6%) [24–26,38]. This variability across settings may be attributed to differences in screening instruments, population characteristics, cultural perceptions of attention-related difficulties, and levels of awareness and stigma surrounding ADHD symptoms. Importantly, the use of self-reported screening tools rather than clinical diagnostic interviews may also contribute to variability in reported prevalence across studies.

From a contextual perspective, the relatively high prevalence observed in this study may reflect the demanding nature of medical education, which is characterized by sustained cognitive load, time pressure, and competitive academic environments [39]. In Lebanon, these academic stressors are further compounded by broader structural challenges, including economic instability, political uncertainty, and reduced access to mental health services, all of which have been shown to negatively affect psychological well-being among young adults [40,41]. Within this context, students with underlying attentional vulnerabilities may be particularly susceptible to symptom exacerbation under chronic stress conditions.

A key finding of this study is the significant independent association between higher ADHD symptom scores and psychological distress, particularly depressive symptoms, followed by poor sleep quality. This relationship may be explained by shared neurobiological mechanisms involving dysregulation of dopaminergic and noradrenergic systems, which are implicated in both attentional regulation and mood and sleep processes. In addition, persistent academic difficulties related to ADHD symptoms may contribute to secondary emotional distress, thereby reinforcing a bidirectional relationship between attentional difficulties, depressive symptoms, and sleep disturbance [42–44]. Consistent with these results, students who screened positive for ADHD reported significantly higher PHQ-9 scores, supporting previous evidence of strong comorbidity between ADHD symptoms and depressive disorders in university populations [25,26,38,45–47]. Poor sleep quality further strengthens this association, as sleep disruption is both a potential consequence and exacerbating factor of attentional and mood dysregulation. This finding is consistent with evidence suggesting that sleep deprivation and irregular sleep patterns can impair executive functioning, attention regulation, and emotional control. Conversely, individuals with ADHD symptoms may experience difficulty initiating and maintaining sleep due to hyperarousal and impaired self-regulation, suggesting a potentially reciprocal relationship between sleep quality and attentional difficulties [48,49].

Importantly, anxiety symptoms were not significantly associated with ADHD scores after adjustment, suggesting that the observed relationship between ADHD symptoms and internalizing psychopathology is primarily driven by depressive rather than anxiety-related features in this population. This finding underscores the importance of distinguishing between different dimensions of psychological distress, particularly when evaluating overlapping symptom profiles in student populations.

Among behavioral factors, smoking was the strongest independent predictor of higher ADHD scores. This association may be explained by neurobiological mechanisms involving dopaminergic reward pathways, where nicotine use may serve as a form of self-medication for attentional deficits and impulsivity. Genetic predispositions affecting dopamine regulation may further contribute to this association, suggesting a complex interaction between biological vulnerability and behavioral coping strategies [50,51].

Clinical and developmental factors also played a significant role. The presence of chronic comorbid conditions was independently associated with higher ADHD scores, suggesting that cumulative health burden may exacerbate cognitive and attentional strain in medical students. Additionally, a history of birth complications was significantly associated with higher ADHD symptom scores, supporting neurodevelopmental models that link early perinatal adversity to later attentional regulation difficulties.

Sociodemographic and academic variables also demonstrated significant associations in the adjusted model. Students in their fourth academic year had significantly higher ADHD scores compared to first-year students. This may reflect increased academic demands, transition to more clinically oriented training, and heightened workload during mid-training periods, which may amplify pre-existing attentional vulnerabilities [52–55]. Furthermore, students from middle socioeconomic backgrounds reported higher ADHD scores compared to those from higher socioeconomic status, suggesting that social determinants may influence symptom expression through differential exposure to stressors, academic resources, or coping capacity [56,57].

Taken together, these findings support a biopsychosocial framework for understanding ADHD symptom expression among medical students. Within this framework, neurodevelopmental vulnerability (e.g., birth complications) interacts with modifiable behavioral factors (e.g., smoking, sleep quality), psychological distress (particularly depression), and academic/environmental stressors to influence the severity of attentional symptoms. This integrated perspective emphasizes that ADHD symptoms in university students should not be viewed in isolation but rather as part of a broader interplay between mental health, behavior, and environmental stress.

From a contextual perspective, the Lebanese setting may further intensify these associations. Medical students in Lebanon are exposed to high academic demands within a broader context of economic instability and psychosocial stressors, which may exacerbate depressive symptoms, sleep disruption, and maladaptive coping behaviors, thereby indirectly contributing to higher ADHD symptom expression [40].

### Practical implications

These findings highlight the importance of comprehensive mental health screening in medical schools that extends beyond depression and anxiety to include ADHD symptomatology and sleep assessment. Early identification of at-risk students could be complemented by tailored interventions such as cognitive-behavioral therapy, time management and organizational skills training, and stress-reduction programs. Academic accommodations, including flexible deadlines, structured study support, and exam modifications, may help students with ADHD manage their workload more effectively. Furthermore, promoting awareness and reducing stigma around ADHD within the medical education environment is crucial, as it encourages students to seek help without fear of judgment. Faculty and administrative staff should also receive training to recognize ADHD-related difficulties and provide appropriate guidance or referrals. Finally, interventions promoting sleep hygiene and smoking-related counseling may help address modifiable factors associated with ADHD symptoms in this population. Collectively, these strategies could not only improve academic outcomes but also enhance psychological resilience and long-term professional success for medical students experiencing ADHD symptoms.

### Strengths and limitations

This study has several strengths. First, it provides multicenter data on probable ADHD symptoms among medical students in Lebanon, a population for which local evidence remains limited. Second, the study used standardized instruments to assess ADHD symptoms, depressive symptoms, anxiety symptoms, and sleep quality, allowing a comprehensive evaluation of factors related to students’ mental health and well-being..

Third, to our knowledge, this is the first Lebanese study to screen for probable ADHD symptoms among medical students while examining a broad range of associated factors, including sociodemographic, academic, clinical, prenatal, lifestyle, and psychological variables. These strengths increase the relevance of the findings and provide a basis for future research and targeted student mental health support programs.

Despite these strengths, this study has several limitations. First, the cross-sectional design precludes causal inference. Second, all variables including ADHD symptoms, psychological distress, prenatal history, and lifestyle factors were assessed using self-report measures, which may have introduced recall bias and social desirability bias. Third, ADHD was assessed using a screening tool rather than a formal clinical diagnostic interview; therefore, the findings should be interpreted as probable ADHD symptoms rather than confirmed ADHD diagnosis or clinical severity. Moreover, the study was conducted among medical students only, which may limit generalizability to other university students or regions. In addition, unmeasured factors such as coping strategies, personality traits, academic performance, and access to university mental health services may have influence the observed associations. The use of snowball sampling and social media-based dissemination may have introduced selection bias, as participation may have been more likely among students who were more active online, more interested in mental health topics, or more psychologically distressed. Finally, information regarding the availability and utilization of university-based mental health services was not collected. Access to such services may influence the recognition, reporting, and management of ADHD symptoms among students and should be explored in future research.

## Conclusion

In conclusion, this study demonstrates that probable ADHD symptoms are relatively common among Lebanese medical students and are independently associated with a range of psychological, behavioral, clinical, and academic factors. The strongest independent associations were observed with depressive symptoms and poor sleep quality, followed by smoking behavior, birth complications, chronic comorbid conditions, academic year, and socioeconomic status. The results underscore the need for targeted mental health strategies in medical schools, including integrated screening for ADHD symptoms alongside depression and sleep problems, early identification of at-risk students, and implementation of tailored interventions such as psychological counseling, sleep hygiene programs, smoking cessation support, and academic assistance for students experiencing functional impairment. Future longitudinal and clinically based studies are needed to confirm these associations and clarify causal pathways.

## Supporting information

S1 Data Participant’s Data. (XLSX)

## References

[1] DSM Library [Internet]. Diagnostic and Statistical Manual of Mental Disorders (DSM) | Psychiatry Online. [cited 2026 Jun 20]. Available from: https://psychiatryonline.org/dsm?doi=10.1176%2Fdsm&publicationCode=dsm

[2] Attention-Deficit/Hyperactivity Disorder (ADHD) - National Institute of Mental Health (NIMH) [Internet]. [cited 2026 Jun 20]. Available from: https://www.nimh.nih.gov/health/statistics/attention-deficit-hyperactivity-disorder-adhd

[3] Newcorn JH, Krone B, Coghill D, Halperin JM. Neurodevelopmental Disorders: Attention Deficit Hyperactivity Disorder (ADHD). Tasman’s Psychiatry. Cham: Springer; 2024. p. 1615–54.

[4] Al-Wardat M, Etoom M, Almhdawi KA, Hawamdeh Z, Khader Y. Prevalence of attention-deficit hyperactivity disorder in children, adolescents and adults in the Middle East and North Africa region: a systematic review and meta-analysis. BMJ Open. 2024;14(1):e078849. doi: 10.1136/bmjopen-2023-078849 38238059 PMC10806616

[5] Richa S, Rohayem J, Chammai R, Kazour F, Haddad R, Hleis S, et al. ADHD prevalence in Lebanese school-age population. J Atten Disord. 2014;18(3):242–6. doi: 10.1177/1087054712445065 22628148

[6] Ghossoub E, Ghandour LA, Halabi F, Zeinoun P, Shehab AAS, Maalouf FT. Prevalence and correlates of ADHD among adolescents in a Beirut community sample: results from the BEI-PSY Study. Child Adolesc Psychiatry Ment Health. 2017;11:20. doi: 10.1186/s13034-017-0156-5 28428817 PMC5393010

[7] Dalsgaard S, Østergaard SD, Leckman JF, Mortensen PB, Pedersen MG. Mortality in children, adolescents, and adults with attention deficit hyperactivity disorder: a nationwide cohort study. Lancet. 2015;385(9983):2190–6. doi: 10.1016/S0140-6736(14)61684-6 25726514

[8] Reinke AL, Stiles K, Lee SS. Childhood ADHD with and without co-occurring internalizing/externalizing problems: prospective predictions of change in adolescent academic and social functioning. J Atten Disord. 2023;27(13):1520–31. doi: 10.1177/10870547231187146 37496457 PMC10552349

[9] Pliszka SR. Comorbidity of attention-deficit/hyperactivity disorder with psychiatric disorder: an overview. J Clin Psychiatry. 1998;59(Suppl 7):50–8. 9680053

[10] Daviss WB. A review of co-morbid depression in pediatric ADHD: etiology, phenomenology, and treatment. J Child Adolesc Psychopharmacol. 2008;18(6):565–71. doi: 10.1089/cap.2008.032 19108661 PMC2699665

[11] Jerome L, Segal A, Habinski L. What we know about ADHD and driving risk: a literature review, meta-analysis and critique. J Can Acad Child Adolesc Psychiatry. 2006;15(3):105–25. 18392181 PMC2277254

[12] O’Callaghan P, Sharma D. Severity of symptoms and quality of life in medical students with ADHD. J Atten Disord. 2014;18(8):654–8. doi: 10.1177/1087054712445064 22582348

[13] Faraone SV, Larsson H. Genetics of attention deficit hyperactivity disorder. Mol Psychiatry. 2019;24(4):562–75. doi: 10.1038/s41380-018-0070-0 29892054 PMC6477889

[14] Aljadani AH, Alshammari TS, Sadaqir RI, Alrashede NOE, Aldajani BM, Almehmadi SA, et al. Prevalence and Risk Factors of Attention Deficit-Hyperactivity Disorder in the Saudi Population: A Systematic Review and Meta-analysis. Saudi J Med Med Sci. 2023;11(2):126–34. doi: 10.4103/sjmms.sjmms_528_22 37252016 PMC10211419

[15] Kundakovic M, Jaric I. The epigenetic link between prenatal adverse environments and neurodevelopmental disorders. Genes (Basel). 2017;8(3):104. doi: 10.3390/genes8030104 28335457 PMC5368708

[16] Walle KM, Askeland RB, Gustavson K, Mjaaland S, Ystrom E, Lipkin WI, et al. Risk of attention-deficit hyperactivity disorder in offspring of mothers with infections during pregnancy. JCPP Adv. 2022;2(2):e12070. doi: 10.1002/jcv2.12070 37431455 PMC10242954

[17] Wiegersma AM, Dalman C, Lee BK, Karlsson H, Gardner RM. Association of Prenatal Maternal Anemia With Neurodevelopmental Disorders. JAMA Psychiatry. 2019;76(12):1294–304. doi: 10.1001/jamapsychiatry.2019.2309 31532497 PMC6751782

[18] Goldman L, Siddiqui EM, Khan A, Jahan S, Rehman MU, Mehan S. Understanding Acquired Brain Injury: A Review. Biomedicines. 2022;10(9):2167. doi: 10.3390/biomedicines10092167 36140268 PMC9496189

[19] Alkhateeb JM, Alhadidi MS. ADHD Research in Arab Countries: A Systematic Review of Literature. J Atten Disord. 2019;23(13):1531–45. doi: 10.1177/1087054715623047 26794672

[20] Bali P, Sonuga-Barke E, Mohr-Jensen C, Demontis D, Minnis H. Is there evidence of a causal link between childhood maltreatment and attention deficit/hyperactivity disorder? A systematic review of prospective longitudinal studies using the Bradford-Hill criteria. JCPP Adv. 2023 Dec;3(4):e12169. doi: 10.1002/jcv2.12169 38054051 PMC10694545

[21] Gnanavel S, Sharma P, Kaushal P, Hussain S. Attention deficit hyperactivity disorder and comorbidity: A review of literature. World J Clin Cases. 2019;7(17):2420–6. doi: 10.12998/wjcc.v7.i17.2420 31559278 PMC6745333

[22] Katzman MA, Bilkey TS, Chokka PR, Fallu A, Klassen LJ. Adult ADHD and comorbid disorders: clinical implications of a dimensional approach. BMC Psychiatry. 2017;17(1):302. doi: 10.1186/s12888-017-1463-3 28830387 PMC5567978

[23] Al-Yateem N, Slewa-Younan S, Halimi A, Saeed SA, Tliti D, Mohammad M, et al. Prevalence of Undiagnosed Attention Deficit Hyperactivity Disorder (ADHD) Symptoms in the Young Adult Population of the United Arab Emirates: A National Cross-Sectional Study. J Epidemiol Glob Health. 2024;14(1):45–53. doi: 10.1007/s44197-023-00167-4 38079098 PMC11043292

[24] Salama RA, Tadross TM, Ammar AR, Manasrah HT, Razack RA, Koya SM, et al. Prevalence and risk factors of adult attention deficit hyperactivity disorder in university students: A study from the United Arab Emirates. Narra J. 2025;5(2):e1950. doi: 10.52225/narra.v5i2.1950 40951462 PMC12425534

[25] Alsafar FA, Alsaad AJ, Albukhaytan WA. Prevalence of adult attention deficit hyperactivity disorder (ADHD) among medical students in the Eastern Province of Saudi Arabia. Saudi Med J. 2024;45(4):397–404. doi: 10.15537/smj.2024.45.4.20230841 38657995 PMC11147571

[26] Sabir H, Khan M, Imran K, Nisa Z-U, Amer SA. The prevalence of undiagnosed attention-deficit/hyperactivity disorder among undergraduate medical students: a survey from Pakistan. BMC Psychiatry. 2024;24(1):845. doi: 10.1186/s12888-024-06182-4 39587585 PMC11587644

[27] Batool F, Khaliq F, Hamid S. Frequency of Attention Deficit Hyperactivity Disorder Symptoms in Medical Students and Associated Factors-A Cross Sectional Study. J Islam Int Med Coll (JIIMC). 2022;17(2):108–13.

[28] Wolraich ML, Hagan JF Jr, Allan C, Chan E, Davison D, Earls M, et al. Clinical Practice Guideline for the Diagnosis, Evaluation, and Treatment of Attention-Deficit/Hyperactivity Disorder in Children and Adolescents. Pediatrics. 2019;144(4):e20192528. doi: 10.1542/peds.2019-2528 31570648 PMC7067282

[29] Visser MJ, Peters RMH, Luman M. Unmet needs of children and young adults with ADHD: insights from key stakeholders on priorities for stigma reduction. J Atten Disord. 2025;29(3):195–206. doi: 10.1177/10870547241297876 39545385 PMC11694544

[30] Maalouf FT, Ghandour LA, Halabi F, Zeinoun P, Shehab AAS, Tavitian L. Psychiatric disorders among adolescents from Lebanon: prevalence, correlates, and treatment gap. Soc Psychiatry Psychiatr Epidemiol. 2016;51(8):1105–16. doi: 10.1007/s00127-016-1241-4 27246607

[31] Assaf M, Rouphael M, Bou Sader Nehme S, Soufia M, Alameddine A, Hallit S, et al. Correlational insights into attention-deficit/hyperactivity disorder in Lebanon. Int J Environ Res Public Health. 2024;21(8):1027. doi: 10.3390/ijerph21081027 39200638 PMC11353674

[32] Younes S, Hajj A, Sacre H, Mourad N, Akel M, Haddad C, et al. Exploring ADHD understanding and stigma: Insights from an online survey in Lebanon. PLoS One. 2024;19(11):e0310755. doi: 10.1371/journal.pone.0310755 39541296 PMC11563464

[33] Lee NYW, Zhang MWB. Systematic review on prevalence of ADHD, possible ADHD or ADHD symptoms in medical students. Front Psychiatry. 2025;16:1684727. doi: 10.3389/fpsyt.2025.1684727 41409340 PMC12706586

[34] Kessler RC, Adler L, Ames M, Demler O, Faraone S, Hiripi E, et al. The World Health Organization Adult ADHD Self-Report Scale (ASRS): a short screening scale for use in the general population. Psychol Med. 2005;35(2):245–56. doi: 10.1017/s0033291704002892 15841682

[35] Kroenke K, Spitzer RL, Williams JBW. The PHQ-9. J Gen Intern Med. 2001;16(9):606–13. doi: 10.1046/j.1525-1497.2001.016009606.x 11556941 PMC1495268

[36] Sawaya H, Atoui M, Hamadeh A, Zeinoun P, Nahas Z. Adaptation and initial validation of the Patient Health Questionnaire - 9 (PHQ-9) and the Generalized Anxiety Disorder - 7 Questionnaire (GAD-7) in an Arabic speaking Lebanese psychiatric outpatient sample. Psychiatry Res. 2016;239:245–52. doi: 10.1016/j.psychres.2016.03.030 27031595

[37] Buysse DJ, Reynolds CF 3rd, Monk TH, Berman SR, Kupfer DJ. The Pittsburgh Sleep Quality Index: a new instrument for psychiatric practice and research. Psychiatry Res. 1989;28(2):193–213. doi: 10.1016/0165-1781(89)90047-4 2748771

[38] Shen Y, Chan BSM, Liu J, Meng F, Yang T, He Y, et al. Estimated prevalence and associated risk factors of attention deficit hyperactivity disorder (ADHD) among medical college students in a Chinese population. J Affect Disord. 2018;241:291–6. doi: 10.1016/j.jad.2018.08.038 30142587

[39] Bergmann C, Muth T, Loerbroks A. Medical students’ perceptions of stress due to academic studies and its interrelationships with other domains of life: a qualitative study. Med Educ Online. 2019;24(1):1603526. doi: 10.1080/10872981.2019.1603526 31007152 PMC6493308

[40] Al-Khalil ZM, El Sheikh WG, Lababidi GH, Shehayeb EO, Ghanime PM, Talih FR, et al. Impact of socioeconomic and political stressors on mental health: a cross-sectional study on university students in Lebanon. BMC Med Educ. 2025;25(1):91. doi: 10.1186/s12909-025-06701-1 39827125 PMC11743072

[41] El Khoury-Malhame M, Bou Malhab S, Chaaya R, Sfeir M, El Khoury S. Coping during socio-political uncertainty. Front Psychiatry. 2024;14:1267603. doi: 10.3389/fpsyt.2023.1267603 38318483 PMC10839968

[42] Del Campo N, Chamberlain SR, Sahakian BJ, Robbins TW. The roles of dopamine and noradrenaline in the pathophysiology and treatment of attention-deficit/hyperactivity disorder. Biol Psychiatry. 2011;69(12):e145-57. doi: 10.1016/j.biopsych.2011.02.036 21550021

[43] Ruhé HG, Mason NS, Schene AH. Mood is indirectly related to serotonin, norepinephrine and dopamine levels in humans: a meta-analysis of monoamine depletion studies. Mol Psychiatry. 2007;12(4):331–59. doi: 10.1038/sj.mp.4001949 17389902

[44] Mohamed SMH, Börger NA, van der Meere JJ. Executive and Daily Life Functioning Influence the Relationship Between ADHD and Mood Symptoms in University Students. J Atten Disord. 2021;25(12):1731–42. doi: 10.1177/1087054719900251 31971050 PMC8404724

[45] Sedgwick-Müller JA, Müller-Sedgwick U, Adamou M, Catani M, Champ R, Gudjónsson G, et al. University students with attention deficit hyperactivity disorder (ADHD): a consensus statement from the UK Adult ADHD Network (UKAAN). BMC Psychiatry. 2022;22(1):292. doi: 10.1186/s12888-022-03898-z 35459116 PMC9027028

[46] Kwak Y-S, Jung Y-E, Kim M-D. Prevalence and correlates of attention-deficit hyperactivity disorder symptoms in Korean college students. Neuropsychiatr Dis Treat. 2015;11:797–802. doi: 10.2147/NDT.S80785 25848277 PMC4376297

[47] Alghamdi WA, Alzaben FN, Alhashemi HH, Shaaban SS, Fairaq KM, Alsuliamani AS, et al. Prevalence and Correlates of Attention Deficit Hyperactivity Disorder among College Students in Jeddah, Saudi Arabia. Saudi J Med Med Sci. 2022;10(2):131–8. doi: 10.4103/sjmms.sjmms_654_21 35602395 PMC9121697

[48] Lim J, Dinges DF. A meta-analysis of the impact of short-term sleep deprivation on cognitive variables. Psychol Bull. 2010;136(3):375–89. doi: 10.1037/a0018883 20438143 PMC3290659

[49] Wajszilber D, Santiseban JA, Gruber R. Sleep disorders in patients with ADHD: impact and management challenges. Nat Sci Sleep. 2018;10:453–80. doi: 10.2147/NSS.S163074 30588139 PMC6299464

[50] Kollins SH, Adcock RA. ADHD, altered dopamine neurotransmission, and disrupted reinforcement processes: implications for smoking and nicotine dependence. Prog Neuropsychopharmacol Biol Psychiatry. 2014;52:70–8. doi: 10.1016/j.pnpbp.2014.02.002 24560930 PMC4004668

[51] van Amsterdam J, van der Velde B, Schulte M, van den Brink W. Causal Factors of Increased Smoking in ADHD: A Systematic Review. Subst Use Misuse. 2018;53(3):432–45. doi: 10.1080/10826084.2017.1334066 29039714

[52] Alatawi Z. Unmasking the hidden struggle behind the white coat: screening adult ADHD symptoms among medical students at the University of Tabuk, Saudi Arabia. Healthcare. 2025;13(13):1528. doi: 10.3390/healthcare13131528 40648553 PMC12248938

[53] Begum N, Pathath AW, Elballah K, Almubireek MA, Irshad S, Ali SI. Prevalence of Adult Attention Deficit Hyperactivity Disorder among Medical Students, King Faisal University. SEEJPH. 2024;:1348–56. doi: 10.70135/seejph.vi.2056

[54] Malau-Aduli BS, Roche P, Adu M, Jones K, Alele F, Drovandi A. Perceptions and processes influencing the transition of medical students from pre-clinical to clinical training. BMC Med Educ. 2020;20(1):279. doi: 10.1186/s12909-020-02186-2 32838779 PMC7446158

[55] Henning C, Summerfeldt LJ, Parker JDA. ADHD and academic success in university students: The important role of impaired attention. J Atten Disord. 2022;26(6):893–901. doi: 10.1177/10870547211036758 34384265 PMC8859654

[56] Russell AE, Ford T, Williams R, Russell G. The Association Between Socioeconomic Disadvantage and Attention Deficit/Hyperactivity Disorder (ADHD): A Systematic Review. Child Psychiatry Hum Dev. 2016;47(3):440–58. doi: 10.1007/s10578-015-0578-3 26266467

[57] Markham WA, Spencer N. Factors that mediate the relationships between household socio-economic status and childhood Attention Deficit Hyperactivity Disorder (ADHD) in children and adolescents: A systematic review. PLoS One. 2022;17(3):e0262988. doi: 10.1371/journal.pone.0262988 35231056 PMC8887716

